# Hospital and Community Ampicillin-Resistant *Enterococcus faecium* Are Evolutionarily Closely Linked but Have Diversified through Niche Adaptation

**DOI:** 10.1371/journal.pone.0030319

**Published:** 2012-02-17

**Authors:** Marieke J. A. de Regt, Willem van Schaik, Miranda van Luit-Asbroek, Huberta A. T. Dekker, Engeline van Duijkeren, Catherina J. M. Koning, Marc J. M. Bonten, Rob J. L. Willems

**Affiliations:** 1 Department of Medical Microbiology, University Medical Center Utrecht, Utrecht, the Netherlands; 2 Department of Infectious Diseases and Immunology, Faculty of Veterinary Medicine, Utrecht University, Utrecht, the Netherlands; 3 Department of Internal Medicine, University Hospital Maastricht, Maastricht, the Netherlands; University of Hyderabad, India

## Abstract

**Background:**

Ampicillin-resistant *Enterococcus faecium* (ARE) has emerged as a nosocomial pathogen. Here, we quantified ARE carriage in different community sources and determined genetic relatedness with hospital ARE.

**Methods and Results:**

ARE was recovered from rectal swabs of 24 of 79 (30%) dogs, 11 of 85 (13%) cats and 0 of 42 horses and from 3 of 40 (8%) faecal samples of non-hospitalized humans receiving amoxicillin. Multi-locus Sequence Typing revealed 21 sequence types (STs), including 5 STs frequently associated with hospital-acquired infections. Genes previously found to be enriched in hospital ARE, such as IS*16*, *orf903*, *orf905*, *orf907*, were highly prevalent in community ARE (≥79%), while genes with a proposed role in pathogenesis, such as *esp*, *hyl* and *ecbA*, were found rarely (≤5%) in community isolates. Comparative genome analysis of 2 representative dog isolates revealed that the dog strain of ST192 was evolutionarily closely linked to two previously sequenced hospital ARE, but had, based on gene content, more genes in common with the other, evolutionarily more distantly related, dog strain (ST266).

**Conclusion:**

ARE were detected in dogs, cats and sporadically in healthy humans, with evolutionary linkage to hospital ARE. Yet, their accessory genome has diversified, probably as a result of niche adaptation.

## Introduction

*Enterococcus faecium* is a common inhabitant of the gastrointestinal tract of humans and animals, frequently causing opportunistic infections in critically ill patients. During the 1980s, the incidence of infections caused by ampicillin-resistant *E. faecium* (ARE) rapidly increased in the U.S., followed by an epidemic rise of vancomycin-resistant *E. faecium* (VRE) in the 1990s [1]–[2]. Nowadays, more than 90% of *E. faecium* recovered from healthcare associated infections in the U.S. are ampicillin-resistant and 80% are vancomycin-resistant [3]. In Europe, the majority of nosocomial invasive *E. faecium* isolates are resistant to ampicillin and VRE infection rates are increasing in several countries [4].

Molecular epidemiological studies based on Multi-locus Sequence Typing (MLST) revealed that the vast majority of *E. faecium* isolates causing clinical infections and nosocomial outbreaks belong to a globally dispersed polyclonal subpopulation, genotypically different from *E. faecium* strains colonizing healthy humans and animals in the community [5]. These so-called hospital *E. faecium* strains, which have been collectively termed Clonal Complex 17 (CC17), are characterized by ampicillin and ciprofloxacin resistance and are specifically enriched with over one hundred genes, including genes encoding for antibiotic resistance and factors with a putative role in colonization and/or virulence [5]–[8]. Recent studies have indicated that isolates from CC17 are not strictly clonally related and that there is considerable genetic diversity among these isolates indicating that they most probably do not constitute a single clonal complex [9].

Until recently, ARE were recovered only sporadically from animals and humans outside the nosocomial environment [10]–[11], rendering resistance against ampicillin a highly specific marker for the hospital *E. faecium* subpopulation. Yet, colonization with *E. faecium* resistant to ampicillin was recently reported among Danish and English dogs [12]. In this report, 76% of the colonized dogs carried ARE isolates with sequence types (STs) that are among the most common ARE lineages causing nosocomial infections. The observed overlap in prevailing STs between dog and infectious ARE isolates, raises the important question whether dogs, and perhaps also other community sources, may serve as a reservoir for ARE colonization and infections in hospitalized patients.

In this study, we extended the search for potential community ARE resources by screening domestic animals including dogs, cats and horses for ARE carriage. In addition, we have tested faecal samples from human volunteers, after exposure to selective antibiotic pressure, for ARE. Recovered community ARE were compared to the known hospital ARE reservoir by MLST, and by performing genetic and phenotypic assays examining antimicrobial susceptibility, ampicillin resistance mechanisms and the presence of genes putatively involved in virulence and/or colonization. Finally, two representative canine ARE isolates were selected for high-quality draft whole genome sequencing allowing a comparative genome analysis with previously sequenced *E. faecium* strains from human origin.

## Materials and Methods

### Samples

To study the occurrence of ARE colonization in non-clinical settings, samples were collected from different Dutch community sources. Rectal swabs were taken from 79 dogs from different regions of the Netherlands and from 42 horses from various stables in the province of Utrecht. In cats, the prevalence of ARE colonization was investigated by taking a swab from faecal samples of 85 cats that were sent to the Utrecht University Faculty of Veterinary Medicine for clinical evaluation for various reasons. Since the animal sampling in this study was minimally invasive and not incriminating nor harmful for the participating animals, ethical approval was not deemed necessary. Yet, all owners, gave verbal informed consent for sampling their pets. In addition, faecal samples from 40 healthy human volunteers, collected as part of a previously described clinical trial which was approved by the medical ethics committee of the University Hospital Maastricht, The Netherlands and for which all volunteers gave written informed consent [13], were screened for ARE-colonization. During this trial all participants received 500 mg of oral amoxicillin twice daily for seven days. To observe whether the use of selective antibiotics induced *in vivo* selection of ARE three faecal samples per participant, collected before (at day 0), during (at day 7) and after (at day 14) antibiotic treatment, were screened for ARE.

### Microbiology and genotyping

The human faecal samples were frozen in a 1∶4 glycerol-peptone dilution at −20°C [13]. Rectal swabs and faecal samples were analyzed for the presence of ARE by inoculating 10 ml of Enterococcosel Enrichment Broth (Becton Dickinson, Cockeysville, MD) supplemented with aztreonam (75 mg/L) with rectal swabs or with 500 µl of the faecal dilution. These enrichment cultures were incubated for 48 hours at 37°C. The samples were subsequently cultured on Enterococcosel Agar plates (Becton Dickinson) supplemented with ampicillin (16 mg/L) for 48 hours. For each ARE-positive sample, one colony was picked for further analyses. All ARE were genotyped using Multi Locus Sequence Typing (MLST) [14] to determine the clonal relatedness among the obtained ARE and with the known nosocomial reservoir.

### Antimicrobial susceptibility

MICs for ampicillin, vancomycin, gentamicin, ciprofloxacin, tetracycline, erythromycin and imipenem were determined in all isolated ARE strains using the Clinical and Laboratory Standards Institute (CLSI) broth dilution method. Strains were classified susceptible, intermediate or resistant for each antimicrobial, based on breakpoints defined by the CLSI or by the European Committee on Antimicrobial Susceptibility Testing (EUCAST; www.eucast.org).

### DNA sequence analysis of *pbp5*

In hospital ARE high-level ampicillin resistance has been linked to mutations in the 3′ region of the *pbp5* gene [15]. To assess whether ampicillin resistance in community strains is caused by the same mechanism, a DNA sequence analysis of *pbp5* was performed. Total DNA was obtained from all recovered ARE as described before [16]. Generated sequences were compared with a *pbp5* gene reference sequence (GenBank accession no X84860).

### Detection of putative virulence genes and DNA elements specifically enriched in hospital and community isolates

By Southern blot analysis, all ARE strains were screened for the presence of the putative virulence genes *esp*, *hyl*, *sgrA*, *ecbA*, *acm*, *sagA*, *pilA* (*fms21*), *pilB* (*ebpC*_fm_), *orf903* (*fms11*), *orf905* (*fms19*), *orf907* (*fms16*) [6], [17], all encoding for adhesins, the gene *orf1481* [18], which is located on a previously identified putative metabolic island, and the IS-element *IS16* [7] using the probes depicted in Table 1 [12]. Of these genes and elements, *esp*, *hyl*, *sgrA*, *ecbA*, *orf903–907* (*fms11*, *16 and 19*), *orf1481* and IS*16* have previously been described as being enriched in hospital ARE [5]–[8], [17], [19]–[20]. In addition, interruption of *acm* by IS*256* was determined by PCR, as the presence of *IS256* in *acm* is negatively associated with hospital ARE [19]. *E. faecium* E1162, an ampicillin resistant blood isolate from a hospitalized patient, and E135, an ampicillin-susceptible faecal isolate from a non-hospitalized person, were included as positive and negative controls, respectively [21].

**Table 1:** Oligonucleotide sequences.

Gene	Probe name	Oligonucleotide sequence (5′→3′)
*esp*	esp14F	AATTGATTCTTTAGCATCTGG
	esp12R	AGATTTCATCTTTGATTCTTGG
*hyl*	hylF	GAGTAGAGGAATATCTTAGC
	hylR	AGGCTCCAATTCTGT
*sgrA*	sgrAF	AATGAACGGGCAAATGAG
	sgrAR	CTTTTGTTCCTTAGTTGGTATGA
*ecbA*	ecbAF	GCAGTTTACAATGGTGTGAAGCAA
	ecbAR	CGGCTAATGAGTATTTGTCGTTCC
*orf903*	903F	TCAACGGACATACCATACCA
	903R	TCAGTTGGATTCCATGTGAT
*orf905*	905F	GTGACAGATTCATCTAATCAT
	905R	TCATTTTATTTCCCTCCTATTG
*orf907*	orf907F	GTGACCGGTTTTGATGAAAAC
	orf907R	TTAAGCTTCTGTTTCTTGATGGC
*acm*	acmF1	GATTTTTGAGATGATGATATAGTAG
	acmR4	GTATCTTCAGGTAGCATGTCTCC
*pilA*	pilAF	TGCTGATTTTGTTGGTATTTCG
	pilAR	GGCGTTCCTGAAGAGAACTCT
*pilB*	pilBF	GTGTTTGCAGAGGAGACAGC
	pilBR	GACAGAATAATTTACTGGGTCG
*sagA*	sagAF	CATGCTGACAGCAAAGTCA
	sagAR	AGAAGCACGCGAACAAGCA
*orf1481*	1481F	GTTTATCAACATGCTAGCCCA
	1481R	GCCAATGAGTTAGATGTAGCC
IS*16*	IS16F	AGCGGTGCGAATGATACCGC
	IS16R	CTTCCGATTCGCCGTCTTGAAC
*EfmE4452_1561*	1561F	CATCGGTACAAGCGGAGTTT
	1561R	TTCCGTTTTCAATGTGACGA
*EfmE4452_1565*	1565F	ATTGTTCGTGCCGGAGATAC
	1565R	GATGATCCCATTCCATTTGC

Putative virulence genes that were found to be less prevalent among the recovered community isolates than previously reported in hospital ARE, were subjected to further analysis. In community isolates with STs frequently observed among nosocomial ARE infections and outbreaks (ST16, ST18, ST19, ST78 and ST192), obtained from this study and the study of Damborg *et al.* [12], the prevalence of *esp*, *hyl*, *sgrA*, *ecbA* and *orf1481* was compared with the occurrence of these genes in hospital ARE isolates with similar STs, using Fisher's Exact test in SPSS 15.0 (SPSS Inc. Chicago, IL, USA). For this comparison hospital ARE strains were recovered from the MLST database (http://efaecium.mlst.net/, queried March 2010) and completed with isolates and additional data extracted from articles retrieved from Medline, that linked the aforementioned STs with the presence of one or more of the genes of interest [22]–[32].

The presence of the genes Efm4452_1561/EfmE4453_1839 and EfmE4452_1566/EfmE4453_1835, which are both contained on a putative mobile genetic element with a predicted role in the breakdown, transport and metabolism of xylopolysaccharides was determined by PCR (see table 1 for primer sequences).

### Genome analysis of two dog isolates

Two representative canine ARE isolates from this study, E4452 and E4453, were selected for whole genome analysis. Chromosomal DNA was isolated as described previously [9] and sequenced on the Illumina Genome Analyzer IIx with a read length of 50 nt according to the manufacturer's protocol. A total of 5056696 and 7397885 matched reads were obtained for E4452 and E4453, respectively, resulting in 91× and 131× genome coverage. Assembly and annotation of the genomes was performed using the CLCbio Genomic Workbench version 3.7 (CLCbio, Aarhus, Denmark). Annotations were subsequently manually curated. The Whole Genome Shotgun projects of strains E4452 and E4453 have been deposited at DDBJ/EMBL/GenBank under the accession AEDZ00000000 and AEOU00000000.

Phylogenomic analysis of *E. faecium* was performed using the amino acid sequences of a set of 500 orthologous proteins of identical length that occur in E4452, E4453 and seven *E. faecium* genomes that were previously sequenced [9]. These sequences were aligned and concatenated using Geneious Pro 4.8.4 and subsequently phylogentic reconstruction was inferred using the Neighbor-Joining method, including bootstrapping with 1000 iterations. To determine differences in gene content between isolates, pairwise comparisons on the set of annotated proteins using BLAT [33] version 33×5 were performed. Proteins were scored as conserved between two strains when bi-directional hits with an amino acid identity ≥90% covering ≥50% of both protein sequences could be identified.

## Results

### ARE carriage

In the cross-sectional screening of different domestic animals, 24 (30%) of 79 dogs, 11 (13%) of 85 cats and none of 42 horses were colonized with ARE. Of the 24 colonized dogs, two lived together in one household while four shared their home with another non-colonized dog. There was no epidemiological link between the colonized cats. One dog was colonized with two morphologically different strains, which were both included for further analysis. ARE was isolated from faecal samples of three (7.5%) of 40 healthy human volunteers that had received oral amoxicillin. In two participants ARE was isolated after antibiotic use (in one at day seven and in the other at both day seven and fourteen). In one participant ARE was only detected in the faecal sample taken before the start of amoxicillin administration.

### MLST

MLST analysis of 39 community ARE strains revealed 21 different STs, including eight new STs (Table 2). ST266 was isolated most frequently and was found in both cats and dogs and in one human volunteer. Comparison with the international *E. faecium* MLST database revealed that four of the recovered 21 STs have been previously cultured from dogs. Eleven of the 21 STs have been isolated from hospitalized patients before, of which nine were associated with clinical infections. Two STs were previously cultured from non-hospitalized persons and three from livestock. MLST analysis revealed that the two colonized dogs sharing a household carried different STs (ST192 and ST373). The two morphologically different strains recovered from a single dog did not have identical STs (ST266 and ST274).

**Table 2:** Multilocus sequence types (STs) of recovered ARE isolates and previous occurrence among other sources.

ARE isolates current study		No. of isolates with identical ST in MLST database^a^ per source					
		Hospital^b^	Community^c^				Total
ST	Frequency (source)	HAI	CS	LS	D	C	All
16	1 (D)	47	-	-	-	-	72
18	1 (C)	103	1	1	-	-	114
19	2 (D)	7	-	-	5	-	12
78	1 (C)	139	1	-	16	-	168
128	1 (D)	1	-	-	-	-	1
148	1 (D)	-	-	1	-	-	1
168	2 (D)	3	-	-	-	-	3
192	2 (D)	56	-	-	8	-	64
264	1 (C)	1	-	-	-	-	1
266	12 (6x C, 5x D, 1x H)	2	-	-	10	-	12
274	4 (1 x C, 3x D)	1	-	-	-	-	1
373	1 (D)	1	-	-	-	-	1
393	1 (H)	-	-	1	-	-	1
453	1 (D)	-	-	-	-	-	-
454	1 (D)	-	-	-	-	-	-
455	1 (D)	-	-	-	-	-	-
456	2 (D, H)	-	-	-	-	-	-
457	1 (D)	-	-	-	-	-	-
458	1 (D)	-	-	-	-	-	-
459	1 (D)	-	-	-	-	-	-
477	1 (C)	-	-	-	-	-	-

a http://efaecium.mlst.net/ queried March 2010;

b HAI = hospital-associated isolates (i.e., clinical isolates, hospital surveillance, hospital outbreak);

c CI = clinical isolate; CS = Community human surveillance; LS = Livestock; D = dogs; C = cats.

### Antimicrobial susceptibility and *pbp5*

All 39 isolates displayed high-level resistance to ampicillin, with MICs ranging from 64 to >512 µg/ml, and resistance to imipinem, with MICs ranging from 8 to 256 µg/ml. In addition, 35 (90%) and 30 (77%) isolates were resistant to tetracycline (MICs ranging from 16 to >64 µg/ml) and erythromycin(all MICs>32 µg/ml), respectively. All isolates were susceptible to vancomycin. High-level resistance to gentamicin (MIC>128 µg/ml) was present in two (5%) strains and high-level resistance to ciprofloxacin (MIC>64 µg/ml), which is associated with hospital ARE [34], was observed in only three (1 dog, 2 cats) isolates. Mutations in the C-terminal region of *pbp5* identical to those previously found in hospital ARE and which are linked to high-level ampicillin resistance [15] were found in all strains (Table 3). In total, 10 different *pbp5* alleles, based on the depicted polymorphisms, were identified. Although the recovered alleles were shared by strains with several STs, *pbp5* allele polymorphisms were highly conserved within isolates with identical STs. For example, 11 of the 12 isolates with ST266, recovered from dogs, cats and a human volunteer had *pbp5* allele 8.

**Table 3:** Polymorphisms in the C-terminal region of *pbp5* a.

Allele	Source^b^	ST	426	461	462	466	466′	470	476	477	485	496	497	499	525	546	558	582	586	629	MIC range
Reference X84860			M	Q	V	S	x	H	A	L	M	N	F	A	E	N	A	G	V	E	
1	D (5x)	19 (2x), 454, 455, 457	I	-	-	-	-	Q	-	-	-	K	-	**T**	D	-	-	-	-	**V**	128–256
2	D (2x)	168 (2x)	-	-	-	-	-	Q	-	-	**T**	K	-	**T**	D	-	-	-	L	**V**	128–256
3	D (4x), C(1x)	274 (4x), 458	-	K	-	-	**S**	Q	-	-	**A**	K	-	**T**	D	-	-	-	L	**V**	≥512
4	D (3x), H (1x)	16, 148, 373, 393	-	K	-	-	**S**	Q	-	-	**A**	K	-	**T**	D	-	-	-	-	**V**	≥512
5	D (1x), H(1x)	456 (2x)	-	-	-	D	**S**	Q	-	-	**A**	K	-	**T**	D	-	-	-	L	**V**	>512
6	C (2x)	477, 266	-	-	-	D	**S**	Q	S	M	-	K	L	**T**	D	-	-	-	L	**V**	>512
7	C (1x)	264	-	-	-	D	**S**	Q	S	-	-	K	L	**T**	D	-	-	-	-	**V**	256
8	C (6x), D (9x), H (1x)	18, 192 (2x), 266 (11x), 453, 459	-	-	-	-	**S**	Q	-	-	**A**	K	-	**T**	D	-	-	-	-	**V**	≥512
9	D (1x)	128	-	-	-	-	**S**	Q	-	-	**T**	K	-	**T**	D	-	-	-	-	**V**	64
10	C (1x)	78	-	-	A	-	**S**	Q	-	-	**A**	K	-	**T**	D	T	T	S	-	**V**	>512

a Amino acid mutations that contribute to ampicillin resistance are indicated in bold [15]. The one-letter abbreviation code is used to denote the amino acids. The – sign indicates no change in amino acid compared to the reference allele.

b Source (and frequency) of the isolates carrying a particular allele: D = dog; C = cat; H = human.

### Prevalence of putative virulence genes and DNA elements enriched in hospital isolates

The genes *orf903* (fms1*1*), *orf905* (fms19), *orf907* (fms16), were highly prevalent in community ARE isolates. This was also the case for IS*16*, which previously has been shown to be enriched in hospital ARE (Table 4) [7], [35]. Other genes previously found to be enriched among hospital isolates were found in about half (*sgrA*, *orf1481*) or only in a few (*esp*, *hyl*, *ecbA*) of the community isolates [6], [8], [17]–[18]. When community strains with STs regularly involved in nosocomial infections and outbreaks (i.e. ST16, ST18, ST19, ST78 and ST192), recovered from this study (n = 7) and the study of Damborg et al. (n = 37) [12], were compared with hospital ARE isolates with similar STs recovered from the online MLST database (n = 377) or from literature (n = 60) [22]–[32], *esp*, *hyl* and *sgrA* were significantly underrepresented in the community strains compared to the hospital strains, while this was not the case for *ecbA* and *orf1481* (Table 5). Integration of IS*256* in *acm*, previously suggested to be indicative for community-origin of strains [19], was only found in three isolates. *pilA*, *pilB*, *acm*, and *sagA* were found in 90% or more of the isolates, which is in concordance with earlier observations that these genes are ubiquitously present in *E. faecium* [19]–[20], [36].

**Table 4:** Prevalence of putative virulence genes.

Gene^a^	No. of isolates (%)	Source^b^
**Adhesins**		
*esp*	1 (3)	C
* hyl*	2 (5)	C
*sgrA*	17 (44)	C, D
*ecbA*	2 (5)	C, D
*orf903*	32 (82)	C, D, H
*orf905*	32 (82)	C, D, H
*orf907*	32 (82)	C, D, H
*acm*	39 (100)	C, D, H
*pilA*	35 (90)	C, D, H
*pilB*	38 (97)	C, D, H
*sagA*	39 (100)	C, D, H
**Sugar metabolism**		
*orf1481*	19 (49)	C, D
**IS-elements**		
IS*16*	31 (79)	C, D, H
IS*256* in *acm*	3 (8)	C, D, H
**Community specific genes tentatively involved in sugar metabolism**		
*EfmE4452_1561*	28 (72)	C, D, H
*EfmE4452_1565*	28 (72)	C, D, H

a The genes *acm*, *pilA*, *pilB* and *sagA* are prevalent among all *E. faecium* strains; the genes *EfmE4452_1561* and *EfmE4452_1565* are uniquely present in community ARE isolates (this paper). All other genes are specifically enriched among hospital isolates [5], [7]–[8], [18]–[19], [52].

b Source of the isolates carrying a particular gene: D = dog; C = cat; H = human.

**Table 5:** Comparison community and hospital isolates with similar STs (ST16, ST18, ST19, ST78 and ST192).

Gene	Community isolates (n = 37)^a^		Hospital isolates (n = 437)^b^		p-value^c^
	tested, n	presence gene, n (%)	tested, n	presence gene, n (%)	
*esp*	37	1 (3)	433	314 (73)	<0.001
*hyl*	37	1 (3)	51	24 (47)	<0.001
*sgrA*	36	15 (42)	23	23 (100)	<0.001
*ecbA*	36	18 (50)	23	16 (70)	0.18
*orf1481*	7	7 (100)	21	20 (95)	0.99

a includes 7 isolates(1x ST16, 1x ST18, 2x ST19, 1x ST78, 2x ST192) recovered in this study and 30 (6x ST19, 16x ST78, 8x ST192) isolates recovered by Damborg *et al.* [12];

b includes 377 isolates (60x ST16, 97x ST18, 7x ST19, 160x ST78, 53x ST192) present in the MLST database at March 16, 2010 and 60 isolates (2x ST16, 9x ST18, 49x ST78) described in literature [22]–[32];

c Fisher's Exact test.

### Genome analysis of *E. faecium* E4452 and E4453

To further characterize the evolutionary links between community and hospital *E. faecium* strains, we determined draft genome sequences of two canine *E. faecium* strains (strain codes E4452 and E4453) that were isolated as part of this study. These strains were isolated in August 2008 from two dogs that were kept separate from each other in different households. According to MLST these strains were assigned to ST266 (E4452) and ST192 (E4453). Both these STs are common among dog strains and may therefore be representative for a significant proportion of dog-associated *E. faecium* strains. In this study 12 of 37 dog strains had ST266 and two ST192, while in the study of Damborg ST266 and ST192 were the third and fourth most common dog ST, respectively (Table 2) [12]. Draft genome sequences of these isolates were determined using Illumina sequencing technology, which, in combination with novel assembly methods, has previously been used to successfully sequence genomes of bacteria [37]–[38] to the draft stage.

**Figure 1:**
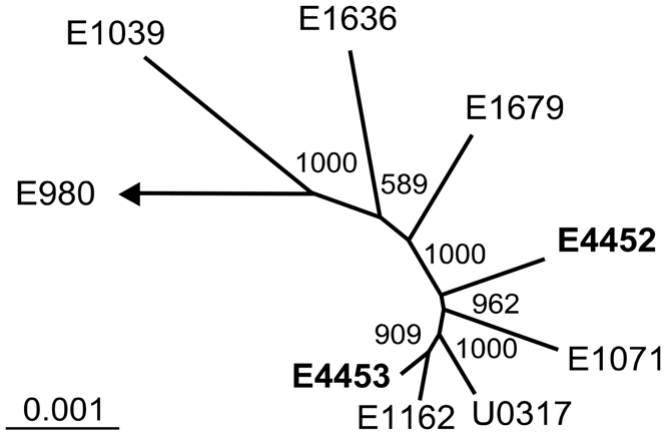
Phylogenomic analysis of canine *E. faecium* strains E4452 and E4453. Unrooted neighbor-joining tree of *E. faecium* based on the concatenated alignments of 500 orthologous proteins (containing 15168 residues). Bootstrap values are based on 1000 permutations.

*De novo* assemblies for both strains resulted in draft genome sequences of E4452 and E4453 containing 2.77 Mbp and 2.82 Mbp, in 268 and 374 contigs, respectively. Contig N50s were 18110 bp and 13956 bp, for E4452 and E4453 respectively. Phylogenomic analysis of the dog strains from this study showed that E4453 (ST192) is relatively closely related to strains E1162 (ST17) and U0317 (ST78) (Figure 1). This is in agreement with the MLST results since ST192 is a single locus variant of ST78 and a double locus variant of ST17. The same analysis demonstrates that strain E4452 (ST266), which based on its MLST profile is not closely related to ST17 and ST78 (3–4 different loci), is more distantly related to the clinical isolates E1162 and U0317 based on phylogenomics.

Interestingly, gene content comparisons between the clinical isolates E1162 and U0317 and the canine strains E4452 and E4453 reveal that the clinical isolates have more genes in common with each than with the two *E. faecium* strains from dogs and, *vice versa*, that the strains from dogs have more genes in common with each other than with the clinical isolates (Table 6). This indicates that there are genes and genetic elements that are specific for either the canine or the clinical strains. Indeed, we were able to identify 32 genes that were shared by both canine *E. faecium* strains but which were absent from all other 28 *E. faecium* strains for which the genome sequence was publicly available in December 2010 (Table 7). None of the strains of which the genomes were previously sequenced have been isolated from dogs. The genes that are unique to the two *E. faecium* strains from dogs include a number of genes that are putatively located on mobile genetic elements (plasmids and/or conjugative transposons) and which have a predicted role in the breakdown, transport and metabolism of xylopolysaccharides. PCR analysis on two of the unique canine genes, *EfmE4452_1561* and *EfmE4452_1565* (Table 7), contained on the putative xylopolysaccharides breakdown, transport and metabolism cluster, demonstrated that both genes were concomitantly present in 28 (72%) of the 39 recovered community ARE isolates, including 18 (72%) of 25 canine, eight (73%) of 11 feline and, interestingly, two (67%) of three human isolates with STs that were also carried by dogs (Table 4). Notably, none of these two genes are present in any of the 28 *E. faecium* genome sequences that were publicly available in November 2011. Most of these genome sequences have been determined from clinical isolates, indicating that this element is relatively scarce in hospital-acquired strains. The incongruence between phylogenomic analysis based on conserved protein sequences from the core genome and genomic relatedness based on gene content strongly indicates that niche-specific adaptation of the accessory genome has occurred in *E. faecium* isolates that inhabit the gastrointestinal tracts of dogs.

**Table 6:** Number of shared Coding Sequences (CDS) between canine *E. faecium* isolates E4452 and E4453 and clinical *E. faecium* isolates E1162 and U0317^a^.

Strains	E4452	E4453	E1162	U0317
**E4452**	**2715**	2234	2108	2133
**E4453**		**2823**	2208	2145
**E1162**			**2694**	2268
**U0317**				**2965**

a The total number of CDS in each genome sequence is indicated in bold.

**Table 7:** Genes from canine *E. faecium* strains E4452 and E4453 which are absent from 28 other *E. faecium* genome sequences^a^.

E4452 locus tags	E4453 locus tags	Annotation
EfmE4452_0533	EfmE4453_2272	hypothetical protein
EfmE4452_0534	EfmE4453_2271	hypothetical protein
EfmE4452_0535	EfmE4453_2270	putative mobilization protein
EfmE4452_0537	EfmE4453_2268	hypothetical protein
EfmE4452_0538	EfmE4453_2267	hypothetical protein
EfmE4452_0539	EfmE4453_2266	hypothetical protein
EfmE4452_0540	EfmE4453_2265	replication initiator protein
EfmE4452_0553	EfmE4453_0769	hypothetical protein
EfmE4452_0595	EfmE4453_1802	hypothetical protein
EfmE4452_0597	EfmE4453_1800	hypothetical protein
EfmE4452_1556	EfmE4453_1844	putative ATP-binding protein
EfmE4452_1557	EfmE4453_1843	related to integrase of Tn552
EfmE4452_1558	EfmE4453_1842	Resolvase/integrase Bin
EfmE4452_1560	EfmE4453_1840	toxin-antitoxin system, toxin component, MazF family
EfmE4452_1561	EfmE4453_1839	D-xylulose kinase
EfmE4452_1563	EfmE4453_1837	transporter permease
EfmE4452_1564	EfmE4453_1836	ABC transporter
EfmE4452_1565	EfmE4453_1835	beta-1,4-xylosidase
EfmE4452_1566	EfmE4453_1834	xylose isomerase
EfmE4452_1567	EfmE4453_2638	xylose operon repressor
EfmE4452_1568	EfmE4453_2637	conserved hypothetical protein
EfmE4452_1773	EfmE4453_0609	heavy metal translocating P-type ATPase
EfmE4452_2486	EfmE4453_2323	hypothetical protein
EfmE4452_2488	EfmE4453_0817	conjugative transposon protein
EfmE4452_2492	EfmE4453_0822	conjugative transposon protein
EfmE4452_2493	EfmE4453_0823	conjugative transposon protein
EfmE4452_2494	EfmE4453_0825	hypothetical protein
EfmE4452_2495	EfmE4453_0826	conjugative transposon protein
EfmE4452_2496	EfmE4453_0827	NLP/P60 family protein, putative cell wall hydrolase
EfmE4452_2500	EfmE4453_0830	hypothetical protein

a black lines indicate that genes are located on different contigs in the draft genome sequences.

## Discussion

The rapid emergence of ARE as important nosocomial pathogen during the last two decades is thought to be the result of intra- and inter-hospital transmission of a relatively limited number of clones with a genetic make-up favouring colonisation, infection and subsequent transmission among hospitalized patients [6]. Possibly, influx of ARE from the community also contributes to its emergence in hospitals, since ARE have been recovered from community sources. In the past decade, ARE carriage was found to be prevalent among dogs and/or cats in Italy, Belgium, Portugal and the U.S. [39]–[42]. Furthermore, ARE had also been isolated from canine urinary tract infections and feline surgical site infections in the U.S. and Switzerland, respectively [43]–[44]. However, since none of these studies determined the genotypic background of ARE isolates, their descent and potential linkage to hospital clones remains unknown. A potential genetic link between ARE in animals and the hospital setting was proposed in 2009 by Damborg and co-workers who described widespread ARE carriage among English and Danish dogs and showed that most of these isolates belong to clones associated with nosocomial infections [12].

Here we present evidence of genotypic concordance, based on MLST, between hospital and community ARE. This demonstrates that community and hospital ARE isolates are evolutionarily linked. The question is whether evolutionary linkage between hospital and community ARE also implies epidemiological linkage, i.e. cross-transmission between the two reservoirs. Evidently, the ARE population recovered from the community is not an exact copy from the circulating reservoir in Dutch hospitals. Eight of the 21 STs found in the community have, up till now, never been reported to colonize or infect patients (http://efaecium.mlst.net/) and the *esp*, *hyl* and *sgrA* genes, which were previously implicated in *E. faecium* virulence, are underrepresented in these community strains. This denotes a significant discrepancy in accessory gene content between hospital and community ARE that includes putative virulence and antimicrobial resistance genes, and indicates that if zoonotic transfer of ARE occurs, it only occurs infrequently.

Phylogenomic analysis of two dog strains from this study and seven previously sequenced *E. faecium* genomes derived from humans [9] only strengthens this notion. Based on its core genome, the ST192 dog isolate was found to be relatively closely related to the two previously sequenced clinical strains (E1162 and U0317) [9]. This leads to the conclusion that on an evolutionary time scale this particular canine isolate is related to the isolates that are currently causing the majority of clinical infections. Congruent with MLST analysis, the ST266 dog strain was more distantly related to the clinical isolates, indicating that not all ampicillin-resistant isolates from the community are closely related to ampicillin-resistant clinical isolates. Yet, when comparing the gene content of these strains, the two dog strains had more genes in common with each other than with the clinical isolates. A number of genes appeared to be specific for either the strains from dogs or the clinical isolates. For example, both E1162 and U0317 carry a 64–68 kb pathogenicity island (ICEEfm1) that contains the *esp* gene [9], [45]–[46], which is involved in biofilm formation [21] and infections in a mouse model [47]–[48]. Both canine *E. faecium* strains E4452 and E4453 are lacking ICEEfm1 and indeed so far *esp* has not been found among ampicillin-resistant canine isolates. On the other hand, 32 genes including a cluster of genes involved in the breakdown, transport and metabolism of xylopolysaccharides, were uniquely present in the dog strains while being absent in all of the other 28 *E. faecium* genome sequences that are currently publicly available were found. This cluster contains a gene annotated as a β-xylosidase which is homologous to genes from *Enterococcus gallinarum* (67% amino acid identity) and *Roseburia intestinalis* (65% amino acid identity), a common anaerobic xylanolytic gut commensal [49]. This finding suggests that *E. faecium* strains from dogs have acquired a genetic element that enable the metabolism of xylose-containing oligo- and polysaccharides. These sugars, which originate from plant materials, are commonly found in commercial dog foods [50] and may thus reflect a metabolic adaptation of *E. faecium* to the canine (and possibly feline) gastrointestinal tract, especially since the majority of the recovered community isolates carried at least two of the genes contained on this element. From three of the 40 healthy human volunteers ARE could be isolated and in two of them only after oral administration of amoxicillin followed by enrichment cultures. One of the volunteers carried a ST that was previously isolated from a clinical infection, but that did not belong to one of the major clones (STs 16, 17, 18, 78, 117, 192, 202, 203) now frequently encountered in hospitals world-wide (http://efaecium.mlst.net/). The low ARE colonization prevalence among healthy humans in the community and the absence of ARE clones currently dominating the nosocomial epidemiology supports the hypothesis that, in hospitalized patients, endogenous selection only plays a minor role in ARE acquisition, relative to cross-transmission [12], [51]. Interestingly, two human volunteers were colonized with STs that were also isolated from domestic animals, including ST266 which was most frequently found among colonized dogs and cats. Moreover, the accessory genome of these human and animal strains was indistinguishable, including the presence of at least two of the newly identified, unique community genes, that are putatively located on the genetic element with a predicted role in the breakdown, transport and metabolism of xylopolysaccharides. This finding suggests that ARE may occasionally be transferred between humans and pets.

Up till now, we can only speculate on why ARE frequently colonize the feline and canine intestinal tract. In humans, ARE colonization is rarely found without prior exposure to selective antibiotics (Table 2) [10]–[11], and when this is not different for domestic animals it would imply that the intestinal microbiota of these pets is frequently challenged by antibiotics, either through therapeutic intake or via other unknown routes. Another possibility is that dogs and cats represent the natural ecological niche for ARE, which makes that they more easily reside (in higher quantities) in the canine and feline than in the human intestinal tract. Perhaps, the mutations in *pbp5* which confer ampicillin resistance in clinical isolates, may even represent the natural *E. faecium* phenotype in the gastronintestinal tract of dogs and cats. If true, this would imply that nosocomial ARE clones have originated and evolved from the animal reservoir. Yet, we cannot rule out the possibility that community and hospital ARE share a common ancestor of other origin, or, that the canine and feline isolates represent early evolutionary descendents of hospital ARE, who in time have lost genetic properties in the absence of selective forces imposed by the nosocomial environment.

In this study, we have demonstrated that the nosocomial and the community reservoir of ARE, present in patients, dogs, cats and sporadically in healthy humans, are evolutionarily linked but that niche separation and adaptation has driven clones onto different evolutionary trajectories resulting in sequential acquisition or loss of adaptive elements including virulence and antimicrobial resistance traits due to selective forces imposed by either the hospital or community environment. This may imply that *E. faecium* hospital clones have originated and evolved from the animal reservoir and that sequential events of zoonotic transfer may have contributed to the diversity in genetic background and accessory genome observed among the polyclonal ARE subpopulation that successfully resides in the nosocomial setting.
